# IMRT in oral cavity cancer

**DOI:** 10.1186/1748-717X-2-16

**Published:** 2007-04-12

**Authors:** Gabriela Studer, Roger A Zwahlen, Klaus W Graetz, Bernard J Davis, Christoph Glanzmann

**Affiliations:** 1 Department of Radiation Oncology, University Hospital Zurich, Rämistrasse 100, 8091 Zurich, Switzerland; 2 Department of Cranio-Maxillofacial Surgery, University Hospital, Zurich, Switzerland

## Abstract

**Background:**

Except for early T1,2 N0 stages, the prognosis for patients with oral cavity cancer (OCC) is reported to be worse than for carcinoma in other sites of the head and neck (HNC). The aim of this work was to assess disease outcome in OCC following IMRT.

Between January 2002 and January 2007, 346 HNC patients have been treated with curative intensity modulated radiation therapy (IMRT) at the Department of Radiation Oncology, University Hospital Zurich. Fifty eight of these (16%) were referred for postoperative (28) or definitive (30) radiation therapy of OCC.

40 of the 58 OCC patients (69%) presented with locally advanced T3/4 or recurred lesions. Doses between 60 and 70 Gy were applied, combined with simultaneous cisplatin based chemotherapy in 78%. Outcome analyses were performed using Kaplan Meier curves.

In addition, comparisons were performed between this IMRT OCC cohort and historic in-house cohorts of 33 conventionally irradiated (3DCRT) and 30 surgery only patients treated over the last 10 years.

**Results:**

OCC patients treated with postoperative IMRT showed the highest local control (LC) rate of all assessed treatment sequence subgroups (92% LC at 2 years). Historic postoperative 3DCRT patients and patients treated with surgery alone reached LC rates of ~70–80%. Definitively irradiated patients revealed poorest LC rates with ~30 and 40% following 3DCRT and IMRT, respectively.

T1 stage resulted in an expectedly significantly higher LC rate (95%, n = 19, p < 0.05) than T2-4 and recurred stages (LC ~50–60%, n = 102).

Analyses according to the diagnosis revealed significantly lower LC in OCC following definitive IMRT than that in pharyngeal tumors treated with definitive IMRT in the same time period (43% vs 82% at 2 years, p < 0.0001), while the LC rate of OCC following postoperative IMRT was as high as in pharyngeal tumors treated with postoperative IMRT (>90% at 2 years).

**Conclusion:**

Postoperative IMRT of OCC resulted in the highest local control rate of the assessed treatment subgroups. In conclusion, generous indication for IMRT following surgical treatment is recommended in OCC cases with unfavourable features like tight surgical margin, nodal involvement, primary tumor stage >T1N0, or already recurred disease, respectively.

Loco-regional outcome of OCC following definitive IMRT remained unsatisfactory, comparable to that following definitive 3DCRT.

## Background

Except for early T1,2 N0 stages, the prognosis for patients with OCC seems to be worse than for carcinoma in other sites of the head and neck (HNC). Many different treatment approaches have been tested over the last two decades [1-23] (interstitial brachytherapy with its excellent results in early stage T1,2 tumors of the mobile tongue or floor of the mouth is not listed, as this does not fall in the category of the patient sample focussed here). In operable patients, adjuvant as well as so called 'neo-adjuvant' concepts have been employed, using several radio-therapeutic schedules in combination with different chemotherapeutic drugs prior to or following surgery. However, loco-regional control in T3,4 and recurrent stages remains unfavourable. In contrast to pharyngeal and laryngeal tumors, loco-regional outcome in OCC is worse when using definitive radio(-chemo)therapy alone.

Loco-regional disease control has a dominant impact on survival, as distant control rates as high as ~90–95 % at 5 years are reported [24].

Intensity modulated radiation therapy (IMRT) technique represents a novel treatment option with a potential capacity for better loco-regional control in inoperable disease. Improved loco-regional outcome following IMRT has been reported for nasopharyngeal [25-28] and oropharyngeal tumors [22,29,30]. Also in hypopharyngeal tumors, a tendency towards better outcome has been described [31]. Published IMRT results related to OCC are confined to two published articles: a series of 27 patients [22], and 29 patients [23], both including mostly postoperative IMRT patients, with resulting 2-year loco-regional control rates of 59% and 78%, respectively. Both authors found a significantly worse LC rate in OCC compared with oropharyngeal tumors.

To assess disease outcome of OCC following IMRT, we analysed 58 consecutively irradiated OCC patients. In addition, a comparison between the IMRT cohort and our historic OCC cohorts treated with (1) surgery alone, (2) definitive three-dimensional conformal radiation therapy (3DCRT), and (3) postoperative 3DCRT was performed.

## Results

### Disease control of the entire OCC cohort

**Figure 1:**
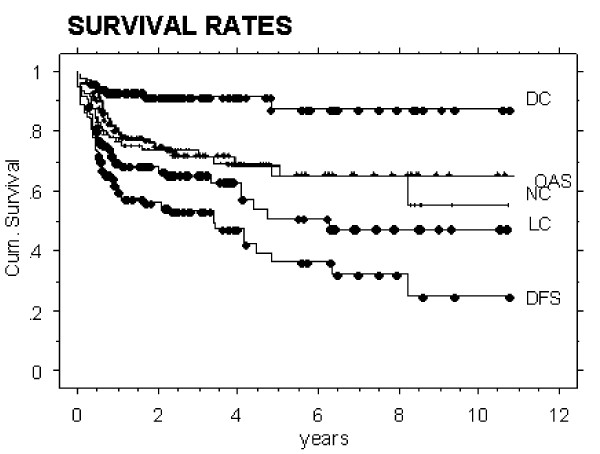
Local (LC), nodal (NC), distant control (DC), overall survival (OAS), and disease free survival (DFS) of the entire analysed oral cancer cavity cohort (N = 121 patients).

Figure 1 shows survival rates of 121 assessed OCC patients treated over the last 10 years (see also Table 1). Eighty % of all loco-regional events have been observed during the first 12 months following treatment.

**Table 1:** Patient and disease characteristics in oral cavity cancer (OCC, N = 121)

**Factors**		**definitive IMRT**	**postop IMRT**	**definitive 3D-CRT**	**postop 3D-CRT**	**Surgery alone**	**N total**
**N**		**30**	**28**	**13**	**20**	**30**	**121**
**Time interval**		10/02 – 1/07	11/02 – 1/07	5/96 – 2/04	04/00 – 3/03	5/96 – 8/05	5/96 – 5/06
**gender **(m:f)		2 : 1	2 : 1	~2 : 1	2 : 1	4 : 1	
**mean age **(years)		61	61	62	60	58	**~60**
**T stages**	T1	0	4	0	2	13	**19**
	T2	6	8	1	5	12	**32**
	T3	3	1	3	1	0	**8**
	T4	12	8	3	10	5	**38**
	recurrence	9	7	6	2	0	**24**
**N stages**	N0	9	7	2	6	19	**43**
	N1	4	4	4	6	7	**25**
	N2a/b	2	12	2	5	2	**23**
	N2c	13	4	1	1	2	**21**
	N3	1	0	0	0	0	**1**
**UICC stages**	l	0	0	0	0	11	**11**
	ll	0	2	0	2	7	**11**
	lll	0	4	5	4	5	**18**
	lVA	20	15	8	14	7	**64**
	lVB	1	0	0	0	0	**1**
	recurrence	9	7	0	0	0	**16**
**concomitant CT**		21/30	24/28	3	0	0	**48**
**mean/median FU **(mo) (range)		16/12 (3–57)	20/19 (4–60)	30/19 (7–96)	40/41 (8–84)	58/48 (16–126)	

FU: follow up; CT: chemotherapy; mo: months

### Outcome according to the treatment modality

The highest LC rate was achieved in patients treated with combined surgery and postoperative IMRT (n = 28, 2-year LC 92%), whereas postoperative 3DCRT (n = 20) and surgery alone (n = 30) resulted in LC rates of ~80%. Definitive radiation reached 2-year LC rates of ~30% following 3DCRT (n = 20) and 43% following IMRT technique (n = 30, p < 0.0005), respectively.

Patients who presented with a recurrence following surgery alone have been analysed separately, as recurrence is characterized by a poor prognosis, with ~30% LC at 2 years.

### Outcome according to the T-stage

**Figure 2:**
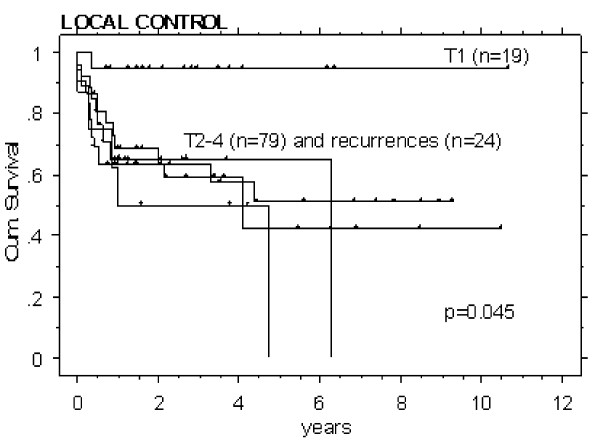
Local control rates of all patients, analysed according to the T-stages. T1 staged tumors showed a superior local outcome (p = 0.045), while all other stages including recurrences, did not differ.

In T1 tumors, a high 2-year LC of 95% (n = 19/121, 13 of them treated with surgery alone, p < 0.05) was found, whereas the LC of T2-4 and recurred tumors showed inferior control rates (~50–60% at 2 years, Figure 2). LC in T1-2 N0-2b stages was found superior to T3-4 N2c and recurred tumors (80 vs 60%, p = 0.01).

In the surgery alone subgroup there were 4 local failures in 14 T1/2 N0 stages (~1/3), one of them with simultaneous nodal relapse, and another two with nodal failure alone (= 6/14 patients with loco-regional failure). When last time seen, four of these 6 patients were alive with no evidence of disease after salvage treatment, two of them were alive with disease.

### Outcome of the IMRT subgroup

The postoperative IMRT subgroup (n = 28) reached 2-year local, nodal, distant control rates of 92, 91, 95%, and disease free and overall survival rates of 87 and 83%, respectively. In the definitive IMRT subgroup (n = 30), the corresponding survival rates were substantially lower with 43, 86, 85, 40, and 30%.

### Outcome of OCC vs pharyngeal tumors treated with IMRT

**Figure 3:**
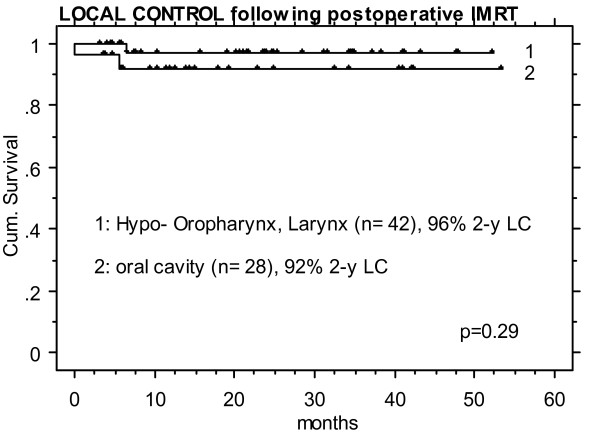
Postoperative IMRT: identically high local control rates in 28 oral cavity cancer patients and 42 patients treated for a squamous cell carcinoma located in the pharynx (nasopharyngeal tumors excluded).

**Figure 4:**
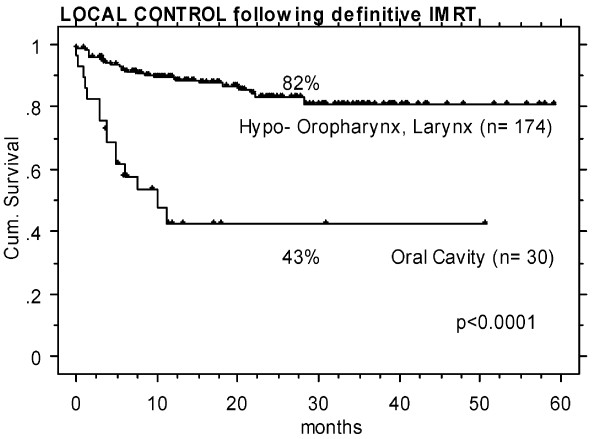
Definitive IMRT: significantly different local control rates in favour to 174 patients treated for squamous cell carcinoma of the pharynx (nasopharyngeal tumors excluded) vs 30 oral cavity cancer (OCC) patients (p < 0.0001) – despite of an identical tumor volume load in the two groups, with mean/median 45/41 cc and 46/39 cc.

Comparisons of LC rates in OCC following postoperative IMRT (n = 28) vs that in squamous cell carcinoma of the oropharynx, hypopharynx, and larynx treated in the same time period (January 2002 to January 2007, n = 42) did not show any significant difference (>90% 2-y LC, p = 0.29, Figure 3), whereas in definitively IMRT irradiated OCC patients (n = 30), LC was significantly worse with 43% vs 82% in definitively irradiated pharyngeal tumors (n = 174, p < 0.0001, Figure 4), despite of a similar volumetric tumor load in these two groups, with total gross tumor volumes of mean/median 45/41 cc in OCC (range 9–123 cc) vs 46/39 cc in pharyngeal tumors (range 1–170 cc).

### Treatment tolerance of IMRT in OCC

IMRT was well tolerated with respect to early toxicity as well as late effects. 14 out of 58 patients needed a temporary gastric feeding tube. No radiation interruption occurred due to treatment related effects. No late xerostomia grade 3 has been observed, and none of these patients at risk for mandible bone necrosis developed a radio-osteonecrosis [32].

## Discussion

The purpose of the current study was to analyse loco-regional disease outcome of OCC following IMRT, related to the outcome of own historic OCC cohorts, in order to assess the value of IMRT in OCC.

The limits of this study are the small size of compared samples, the retrospective character and different treatment intervals and follow up periods of the historic controls, respectively. The different treatment approach with respect to the systemic therapy may, in addition, influence the outcome.

However, the IMRT subgroup data were prospectively assessed and represent the largest OCC IMRT population reported so far.

T1 stage (mainly surgically treated) could be confirmed as a statistically significant favourable outcome predictor. In intermediate and advanced stages, loco-regional control after radiation alone (+/- chemotherapy) is unsatisfactory, and IMRT technique does not seem to have an impact on this fact. Patients with loco-regionally extended disease are often candidates for primary radiation – the definitive radiation group represents per se an unfavourable selection (Table 1); however, in pharyngeal tumors characterized by this same condition, primary radiation is able to reach much higher LC rates, sometimes even approximating those of surgical cohorts. The reason for this difference between OCC and other HNC entities remains speculative; biological differences may likely represent a relevant factor. However, the excellent results following interstitial brachytherapy for early T1,2 N0 stages with LC rates > 80% [2,33,34] prove radiation is basically highly effective in this entity as well, at least for small tumor volumes.

Our T1,2 N0 surgery alone cohort developed loco-regional failure in nearly half the cases, however the sample size is too small to allow to draw reliable conclusions. Surgery combined with postoperative IMRT +/- chemotherapy achieved high loco-regional control rates, also in tumors with intermediate or loco-regionally advanced stages. This observation may be the key information of the current analysis. In addition, postoperative IMRT showed a tendency towards better local control than postoperative 3DCRT, however the sample sizes are small, and this observation needs to be confirmed based on larger sample sizes and longer follow up.

Comparison of the presented OCC results with published data [1-23] is difficult, as too many different factors (like treatment sequence, stage, combined modalities, sample sizes, outcome parameters) confound the results.

To our knowledge two other articles on IMRT in OCC [22,23] are available to date. Eisbruch et al [22] found identical LC rates in postoperative vs definitive IMRT patients, with a significantly better 3-year loco-regional control in oropharyngeal tumors than in the other HNC sub-sites (94% for 80 oropharynx, 75 and 60% for 12 hypopharyngeal and 11 laryngeal tumor patients, and 59% in 27 mostly operated OCC patients, respectively). Similarly, Yao et al [23] observed identical postoperative and definitive IMRT results with respect to LC, and a significantly higher LC rate for their mostly definitively irradiated oropharyngeal tumors (98% 2-y LC vs 78% for mostly operated OCC).

## Conclusion

The following conclusions can be drawn from the presented data:

- Combined treatment with surgery and postoperative (chemo-)IMRT resulted in a high control rate of >90% in OCC >T1N0, comparable to the favourable results in other advanced HNC entities treated with IMRT +/-surgery.

- LC in OCC following definitive IMRT was substantially lower than following postoperative IMRT

- LC in OCC following definitive IMRT was substantially lower than that observed in definitively IMRT-treated pharyngeal tumors with comparable tumor load

- IMRT seems not to improve the unsatisfactory loco-regional outcome in definitively irradiated OCC compared to patients treated with 3DCRT techniques

These findings are, in consequence, suggestive for a combined approach with surgery followed by postoperative IMRT may represent the treatment of choice in OCC >T1 N0. An additional reason for favouring a sooner application of postoperative IMRT is the improved tolerance profile such as substantially reduced xerostomia [22,26,35,36] and a minimized risk for radio-osteonecrosis [32] following IMRT.

## Methods

### Patients

In Table 1, patient and disease characteristics of the entire OCC patient cohort treated over the last decade (5/1996-1/2007) are displayed.

Approximately half the patients presented with a floor of the mouth carcinoma, one third with a tongue/floor of the mouth cancer, 10% with a tumor of the gingival/mandible. The remaining 10% consisted of tumors of the tongue or upper jaw.

#### Assessed subgroups

##### a) IMRT patients

Fifty eight consecutive patients with OCC were irradiated with IMRT at the Department of Radiation Oncology, University Hospital Zurich, between October 2002 and January 2007. 40/58 patients presented with locally advanced T4/3 or recurred disease. Thirty patients (52%) underwent definitive radiation therapy. In 78% of all, simultaneous cisplatin chemotherapy was given.

##### b) 3DCRT controls

Thirty four control patients treated with 3DCRT in the time interval between May 1996 and March 2003 (prior to the clinical implementation of IMRT and the inclusion of all HNC patients in our IMRT program, respectively), were retrospectively assessed for comparative purposes (Table 1). This subgroup was comparable with the IMRT subgroup in terms of T-stages (75% T3,4 or recurred tumors), definitively irradiated patients (~50%), age (~60 y) and gender (2:1), respectively.

##### c) Surgical controls

In addition, 30 consecutive patients who were treated with surgery alone between May 1996 and August 2005, were retrospectively assessed for comparative purposes (Table 1). The percentage of locally advanced T3,4 or recurred cases was expectedly low with 17% (nine patients presented with stage T1N0, 5 with T2N0).

### IMRT Planning systems

Volume delineation, dose calculation and plan optimization was performed on a Varian Treatment Planning System (Eclipse^®^, Version 7.3.10, Varian Medical Systems, Hansen Way, Palo Alto CA, 94304-1129).

### Chemotherapy

Simultaneous chemotherapy was given in most (78%) of the IMRT patients. In the postoperative situation this was not the standard treatment until approximately 2000 [37-39]. Since then, all definitive as well as postoperative patients with no specific contraindications undergo combined simultaneous cisplatin chemotherapy (40 mg/m^2^, 1x/radiation week) at our institution.

### Irradiation

General indications for postoperative radiation in operated patients were locally advanced stages, positive surgical margins, involvement of 2 or more lymph nodes, or extra-capsular extension, respectively.

*-IMRT *was delivered by 6 MV photon beams on a Varian linear accelerator with sliding window technique. The technical solution of choice was a 5 field arrangement ('class solution') for all patients. 70 Gy in 33 sessions was given for definitive IMRT. IMRT treatment was delivered using simultaneously integrated boost (SIB) technique; details on SIB are reported elsewhere [36]. The dose in electively irradiated regions was 54 Gy/33 fractions (range 50–56).

The high dose planning target volume (PTV1): included the gross tumor volume (GTV) and a margin of approximately 1.5 cm. Elective irradiation of lymphatic regions in T3,4 or N1 situations included level I,II,III and lV bilaterally of the neck and level 5 on the ipsilateral side. In patients with N1, the retropharyngeal nodes bilaterally were also included. On the uninvolved side of the neck, the upper field border was at the lower border of the transverse process of C1.

Patient alignment was checked before each irradiation by portal imaging; deviations of >3 mm were corrected before treatment.

*-3DCRT *treatment has been delivered by 6MV photon beams on the same Varian linear accelerator, using standard techniques as described in G Fletcher 1980/WM Mendenhall 1994 (Textbooks).

Definitive 3DCRT has been delivered using accelerated schedules with concomitant boost or standard fractionation with 2.0 Gy per fraction, 6 fractions/week, respectively.

Total treatment doses ranged between 68 and 74 Gy in definitive 3DCRT, and between 60 and 66 Gy in postoperative patients, for IMRT as well as 3DCRT techniques, respectively.

### Statistics

All our statistical analyses consisted of comparing groups according to a time-to-event endpoint (survival analysis), using Kaplan-Meier curves and log-rank tests implemented in StatView^® ^(Version 4.5). P values < 0.05 were considered as significant.

## Competing interests

The author(s) declare that they have no competing interests.

## Authors' contributions

GS and CG designed the study. GS drafted the manuscript.

RZ collected and analysed the surgical cohort, and reviewed the manuscript.

CG, BD, KG and UL reviewed and corrected the manuscript. All authors read and approved the final manuscript.
